# Predicting Habitat Suitability and Range Dynamics of Three Ecologically Important Fish in East Asian Waters Under Projected Climate Change

**DOI:** 10.3390/biology14111476

**Published:** 2025-10-23

**Authors:** Ifeanyi Christopher Nneji, Winnie Wanjiku Mambo, Zhao Zheng, Segun Olayinka Oladipo, Hancheng Zhao, Wentao Lu, Lotanna Micah Nneji, Jianqing Lin, Wenhua Liu

**Affiliations:** 1 Guangdong Provincial Key Laboratory of Marine Disaster Prediction and Prevention, Guangdong Provincial Key Laboratory of Marine Biotechnology, Institute of Marine Sciences, Shantou University, Shantou 515063, China; 20nnejiifeanyi@stu.edu.cn (I.C.N.); 19zzheng@stu.edu.cn (Z.Z.); 20hczhao@stu.edu.cn (H.Z.); 23wtlu@stu.edu.cn (W.L.); 2 International Joint Research Center for Marine Ecological Protection and Disaster Prevention, Shantou University, Shantou 515063, China; 3 Department of Biology, Howard University, Washington, DC 20059, USA; winnie.mambo@bison.howard.edu (W.W.M.); segunolayi.oladipo@howard.edu (S.O.O.); lotannamicah.nneji@howard.edu (L.M.N.)

**Keywords:** climate change, east Asia, ecological niche modeling, marine protected areas, distribution shifts, fisheries

## Abstract

**Simple Summary:**

Climate change poses a significant threat to ecologically important fish species, underscoring the need to predict potential shifts in their distributions. Using ensemble species distribution models based on occurrence data from GBIF and OBIS, we assessed the current and future distributions of *Collichthys lucidus*, *Konosirus punctatus*, and *Clupanodon thrissa* in East Asia under present and future climate scenarios. Key environmental predictors were dissolved oxygen and salinity for *C. lucidus* and chlorophyll and phosphate for *K. punctatus* and *C. thrissa*. Projections indicated a contraction of suitable habitats for *C. lucidus*, in contrast to range expansions for *K. punctatus* and *C. thrissa*. Given the limited protection of these species by existing marine protected areas (MPAs), our findings highlight the urgent need for adaptive conservation strategies, including the expansion and reconfiguration of MPAs to safeguard future habitats.

**Abstract:**

The vulnerability of ecologically important fish species to climate change underscores the need to predict shifts in their distributions and habitat suitability under future climate scenarios. In this study, we modeled the potential distribution ranges of three ecologically important fish species (*Collichthys lucidus*, *Konosirus punctatus*, and *Clupanodon thrissa*) across East Asia using a species distribution modeling framework under both current and projected future climate scenarios. Occurrence data were obtained from the Global Biodiversity Information Facility (GBIF) and the Ocean Biodiversity Information System (OBIS), while environmental data were retrieved from the Bio-ORACLE database. Our models demonstrated high predictive performance (AUC > 0.88). Results showed that dissolved oxygen and salinity were the strongest bioclimatic predictors for *C. lucidus*, whereas chlorophyll and phosphate primarily shaped the distributions of *K. punctatus* and *C. thrissa*. Model projections indicated a decline in suitable habitats for *C. lucidus*, particularly under high-emission scenarios, and range expansions for *K. punctatus* and *C. thrissa* toward higher latitudes and nutrient-enriched waters. Highly suitable habitats were concentrated along coastlines within exclusive economic zones, exposing these species to significant anthropogenic pressures. Conservation gap analysis revealed that only 7%, 2%, and 6% of the distributional ranges of *C. lucidus*, *C. thrissa*, and *K. punctatus*, respectively, are currently encompassed by marine protected areas (MPAs). Our study further identified climatically stable regions that may act as climate refugia, particularly for *C. lucidus* in the Yellow and East China seas. Our findings highlight the urgent need for adaptive management, including the expansion and reconfiguration of MPAs, transboundary conservation initiatives, stronger regulation of exploitation, and increased public awareness to ensure the resilience of fisheries under future climate change.

## 1. Introduction

Aquatic ecosystems support globally significant fisheries but are increasingly threatened by anthropogenic activities, including overfishing, pollution, and habitat degradation [1,2]. Climate change further intensifies these pressures by altering environmental conditions, thereby creating uncertainty for the diversity of wild fish and the sustainability of global fisheries [3,4,5,6]. The impacts of climate change, including rising temperatures, ocean acidification, deoxygenation, and sea-level rise, directly affect marine productivity [7]. These changes often trigger biological responses, including altered growth rates and phenology [8,9], shifts in species distribution due to migration [10,11], and local or widespread extinctions [4,12], all of which undermine the long-term sustainability of fisheries [13,14]. Therefore, understanding the influence of bioclimatic variables on habitat suitability and predicting climate-driven distributional shifts under multiple scenarios and timeframes is critical. Such insights are crucial for identifying conservation gaps and developing strategies to mitigate the impacts of climate change on fisheries and aquatic ecosystems [3,15].

Asia has recorded one of the highest numbers of climate-related disasters globally, resulting in substantial economic losses [16]. This region is particularly vulnerable to climate change because its vast low-lying coasts and river deltas face rising seas and land subsidence, which together increase the risks of storm surges and flooding [17,18]. As a result, Asia has witnessed marked climatic shifts over the past decade, with projections indicating greater severity in the decades ahead, underscoring the urgency for adaptive strategies [19]. These climatic changes have led to declines in net primary production, intensified ocean warming and coral bleaching, and decreased marine biomass and productivity [20]. Consequently, they are reshaping marine habitats, altering fish migration routes, and disrupting key ecological services [21,22], with cascading impacts on marine biodiversity across the region [23,24,25]. Given the region’s strong human dependence on fisheries [24], it is essential to understand how climate change affects fishery resources in Asia.

In Asia, particularly in East Asia, numerous fish species are of substantial economic and ecological importance but are increasingly threatened by the impacts of climate change [25]. Among these species are *Collichthys lucidus* (Richardson, 1844), *Clupanodon thrissa* (Linnaeus, 1758), and *Konosirus punctatus* (Temminck & Schlegel, 1846), which are commercially valuable trawl-caught fish in East Asian fisheries [26,27,28]. These species not only contribute to regional food security but also play critical ecological roles by occupying intermediate trophic levels as planktivorous consumers and serving as essential prey for higher predators such as the Pacific humpback dolphin (*Sousa chinensis* Osbeck, 1765) in marine ecosystems [26,27,28]. However, their populations are currently in decline due to overfishing and ecosystem degradation [29]. Biologically, *C. lucidus* is a small demersal fish of the family Sciaenidae that inhabits coastal benthic zones. It matures at around 80 mm, lives up to three years, and is an economically valuable species across East Asia [30,31,32]. In contrast, *C. thrissa* and *K. punctatus* are pelagic members of the family Clupeidae, both with lifespans of up to five years, and contribute significantly to regional fisheries [33,34]. Despite their ecological and economic importance, studies on the impacts of climate change on these species in Asia remain limited [25,35].

Studies examining the impacts of climate change have increasingly employed species distribution models (SDMs) to estimate relationships between species occurrences and environmental predictors [36,37]. These models are powerful tools for projecting changes in species distributions under future climate scenarios [38]. Widely applied in marine research, ecological models can help identify vulnerable regions and populations while also quantifying the ecological processes that shape species occurrences [39,40]. They are also useful for mapping current and future niches of fish species in the context of climate change and conservation planning [41]. Moreover, recent studies have highlighted the integration of SDMs into marine spatial planning and fisheries management, emphasizing their value in supporting ecosystem-based management and informed decision-making under changing environmental conditions [42]. These applications underscore the vital role of SDMs in assessing and managing fishery resources in a changing climate.

In this study, we assessed the impacts of climate change on the present and projected distributions of three ecologically important fish species (*C. lucidus*, *C. thrissa*, and *K. punctatus*) in East Asia under the three climate scenarios (SSP1-2.6, SSP3-7.0, and SSP5-8.5). Using species occurrence records for each focal species and environmental variables, our specific objectives were to: (i) identify the most influential predictors of suitable habitats under different climate change scenarios; (ii) evaluate the impacts of climate change on species distributions for two future time periods (2050–2060 and 2090–2100); (iii) project climate-driven distributional shifts under three emission scenarios across both future periods; and (iv) conduct a conservation gap analysis to inform long-term management and protection strategies. We predict that SDMs will identify the key environmental factors that determine habitat suitability for the target species [42]. Consistent with previous studies [39,40], we predict that *C. lucidus*, *C. thrissa*, and *K. punctatus* will undergo poleward or depth-related shifts, moving toward cooler, higher-latitude, or deeper marine zones. Furthermore, the conservation gap analysis is expected to reveal a growing proportion of suitable habitats that fall outside existing marine protected areas, underscoring emerging priorities for conservation planning and policy development. Overall, our study provides projections of habitat suitability, distributional shifts, and conservation status for three ecologically important fish species under different climate scenarios and identifies key areas for conservation prioritization.

## 2. Materials and Methods

### 2.1. Species Occurrence Data

**Figure 1:**
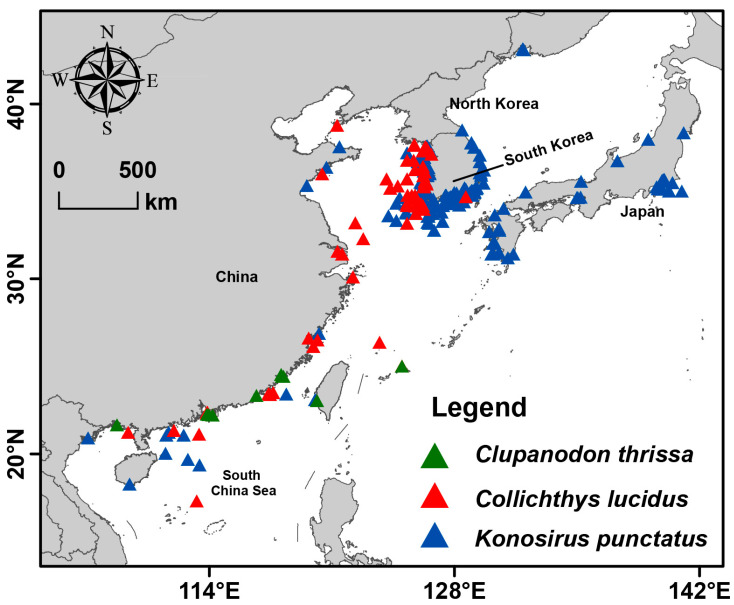
The geographic distribution of three economically important fish in East Asia.

Our study focused on the distribution ranges of *C. lucidus*, *C. thrissa*, and *K. punctatus* in East Asia, particularly within the waters of China, South Korea, and Japan. Occurrence records for these species were obtained from the Global Biodiversity Information Facility (GBIF; https://www.gbif.org/occurrence/; accessed on 13 June 2025) and the Ocean Biodiversity Information System (OBIS; https://obis.org/search/; accessed on 15 June 2025. Data from both sources were merged and subsequently checked and cleaned prior to downstream analyses. Cleaning involved the removal of duplicate records, exclusion of points located outside the study area, and elimination of other erroneous or outlier occurrences. To minimize spatial sampling bias, we applied spatial thinning using the spThin R package, version 0.2.0 [43], ensuring that only a single occurrence point was retained within a 1 km radius. After this procedure, we obtained a total of *n* = 92 for *C. lucidus*, *n* = 355 for *K. punctatus*, and *n* = 19 unique occurrence points for *C. thrissa* (Figure 1), which were used in subsequent analyses. Finally, the cleaned datasets were visualized in ArcGIS 10.8 (ESRI, Redlands, CA, USA) under the World Geodetic System 1984 (WGS84) geographic coordinate system.

### 2.2. Environmental Data

We used environmental data from the Bio-ORACLE database (https://www.bio-oracle.org/, accessed on 13 June 2025)) to predict the potential distribution of the three focal species. Present-day variables (2010–2020) and future projections under three climate scenarios (SSP1-2.6, SSP3-7.0, and SSP5-8.5) were obtained for two time periods: mid-century (2050–2060) and end-century (2090–2100). Future environmental layers represented the ensemble mean of several CMIP6 global climate models, including CanESM5, CESM2-WACCM, GFDL-ESM4, IPSL-CM6A-LR, MIROC-ES2L, MPI-ESM1-2-LR, MRI-ESM2-0, UKESM1-0-LL, ACCESS-ESM1-5, CNRM-ESM2-1, and GISS-E2-1-G [44]. Because *C. lucidus* is a benthic species, we used 16 benthic environmental layers for its analysis (Table S1). In contrast, *K. punctatus* and *C. thrissa* are pelagic species; therefore, 24 ocean surface layers were applied (Table S2). All variables were obtained at a spatial resolution of 0.05°.

**Figure 2:**
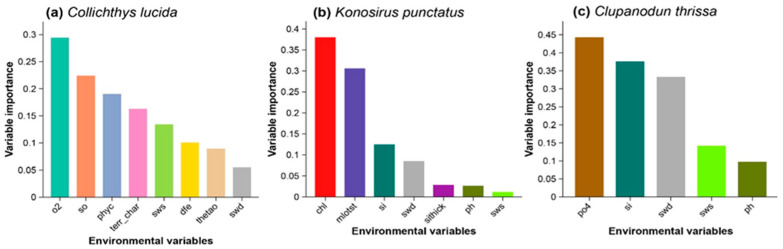
The relative influence of environmental factors on the potential distribution of (**a**) *Collichthys lucidus*, (**b**) *Konosirus punctatus*, and (**c**) *Clupanodun thrissa* in East Asia, as derived from the ecological niche models. Different colors represent the various environmental variables. Note: O_2_ = Dissolved oxygen; so = Salinity; phyc = Phytoplankton concentration; terr_char = Temperature at certain depth/climatological habitat range (likely sea surface temperature or bottom temperature variable used in SDMs); sws = Sea water salinity (surface); dfe = Sea floor depth/current (bathymetry-related); thetao = Potential temperature of seawater and swd = Sea water depth.

Selecting an appropriate calibration region is a crucial step in ecological niche modeling (ENM) and significantly impacts model outcomes [45]. Following the approach of Diaz-Carballido et al. [46], calibration areas for our focal fish species were defined using the Marine Ecoregions of the World (MEOW; Spalding et al. [47]). Only MEOW regions containing at least one occurrence record were included. Since Bio-ORACLE provides global environmental data, variables were cropped to match the shapefile of the calibration area for each species. To reduce model overfitting, we performed multicollinearity analysis of environmental predictors using the vifstep function in the usdm R package [48]. Highly correlated variables (VIF > 5) were excluded. After filtering, eight variables were retained for *C. lucidus*, seven for *K. punctatus*, and five for *C. thrissa*, which were subsequently used in the ENM analysis (Figure 2; Tables S1 and S2).

### 2.3. Ecological Niche Modeling (ENM)

In recent decades, numerous models have been developed to analyze relationships between species distributions and environmental variables [49]. In this study, we used the cleaned distribution data (Figure 1) and selected environmental predictors (Figure 2) for each species to perform ecological niche modeling (ENM). Model calibration, a critical step for optimizing predictive performance, was first conducted. Presence records for each species were partitioned into five folds using the *blockCV* package, version 3.1-4 [50] to reduce spatial autocorrelation and overfitting. Maxent parameters were fine-tuned by testing 17 regularization multipliers (RMs) and all 31 possible feature class combinations (Table S3) using the *kuenm* R package, version 1.1.10 [51]. Optimal parameter settings for each species were reported in Table S4. Model performance was assessed using partial ROC [52], omission rates (OR), and the Akaike Information Criterion corrected for small sample sizes (AICc).

To minimize uncertainties associated with single-model predictions [53], we employed an ensemble approach using five algorithms implemented in the *sdm* R package, version 1.1.8 [54]: the Maximum Entropy (Maxent), Generalized Linear Model (GLM), Random Forest (RF), Boosted Regression Trees (BRT), and Support Vector Machine (SVM). For each species, 1000 pseudo-absence points were generated within the calibration area using the *randomPoints* function of the *dismo* package, version 1.3-9 [55]. We applied 10 bootstrap replicates to randomly partition occurrence and background data into training (70%) and testing (30%) subsets.

Model performance was evaluated using two complementary metrics: the threshold-independent area under the receiver operating characteristic curve (AUC) and the threshold-dependent True Skill Statistic (TSS). AUC values range from 0 to 1, with >0.9 indicating excellent performance, 0.8–0.9 good, 0.7–0.8 fair, and <0.5 worse than random [56,57]. TSS values range from −1 to 1, with values close to 1 denoting high accuracy and values < 0.5 indicating poor predictive power [58]. Only models with AUC ≥ 0.8 were used in the ensemble modeling to ensure the robustness of our results. These models were combined using a weighted average approach based on their AUC values.

Ensemble models were used to predict the distribution ranges of our focal fish species under current conditions and six future scenarios across three climate pathways (SSP1-2.6, SSP3-7.0, and SSP5-8.5). Predictions were generated for two projection periods: mid-century (2050–2060) and late century (2090–2100). All analyses were conducted in R, and output raster layers (GeoTIFF format) were exported to ArcGIS 10.8 (ESRI, Redlands, CA, USA) for visualization. Using the natural break method in the *Reclassify* tool in ArcMap, the predicted suitability models were classified into three categories as follows: (a) highly suitable, (b) moderately suitable, and (c) low suitability.

### 2.4. Projecting Distribution Shift Under Different Climate Scenarios

**Figure 3:**
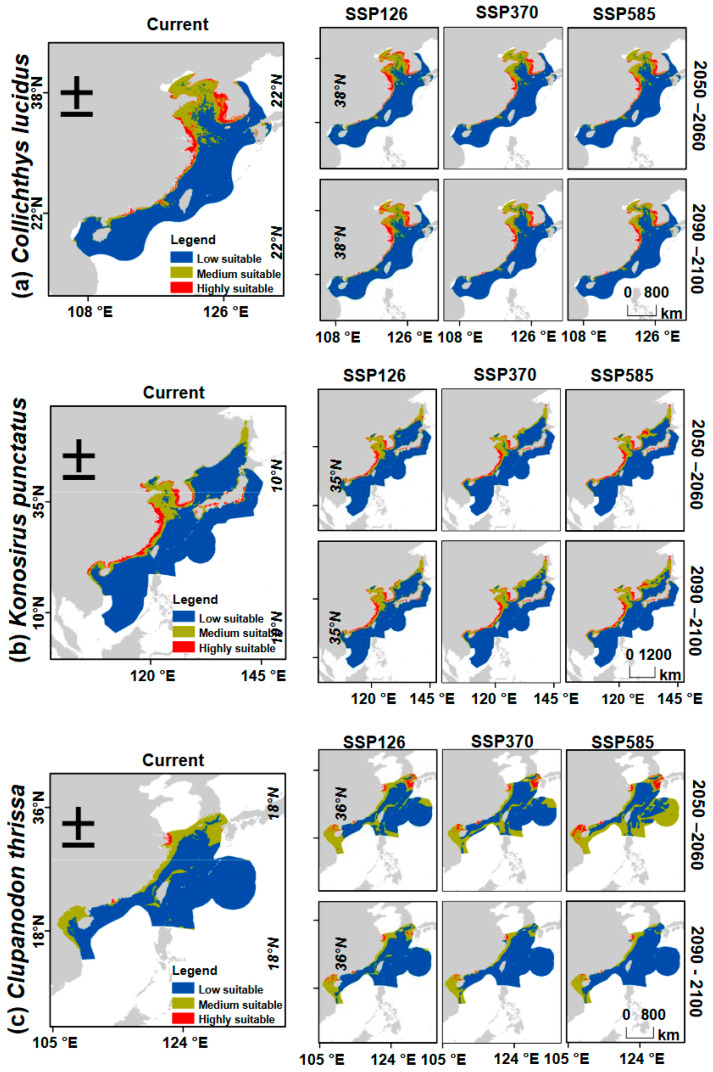
Predicted potential suitable habitats under different climate scenarios for (**a**) *Collichthys lucidus*, (**b**) *Konosirus punctatus*, and (**c**) *Clupanodun thrissa*. The current and three future scenarios (SSP126, SSP370, and SSP585) for the years 2050–2060 and 2090–2100. The grey on the maps denotes the terrestrial regions, while the white areas are the marine areas outside the calibration region.

**Figure 4:**
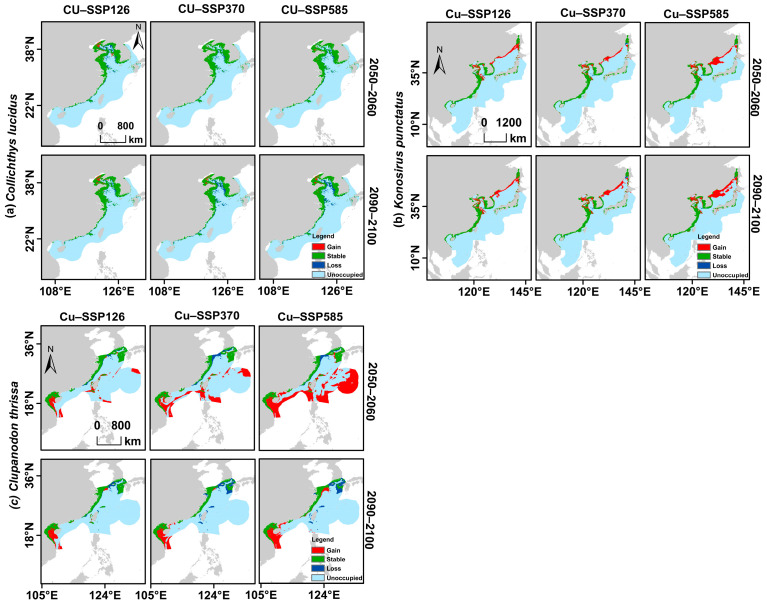
Shifts in the distribution ranges of the three focal fish species under different climate scenarios (**a**) *Collichthys lucidus*, (**b**) *Konosirus punctatus*, and (**c**) *Clupanodun thrissa*. The three future scenarios are SSP126, SSP370, and SSP585 for the years 2050–2060 and 2090–2100.

The ecological niche models obtained from the ensemble analysis for the present and future periods (Figure 3) were used to assess projected changes in the distribution ranges of the focal fish species. To delineate suitable versus unsuitable habitats, we applied the Reclassify tool in ArcMap to generate binary habitat maps using the threshold value for each climate scenario obtained after the ENM analysis. Distributional changes were then quantified using the BIOMOD Range Size function in the biomod2 R package, version 4.2-3 [59], which calculates area change by subtracting the current binary raster from each of the future binary raster layers. The resulting area-change rasters were classified into four categories: stable, loss, gain, and unoccupied (Figure 4). Regions identified as stable habitats across time periods were interpreted as priority conservation areas and potential climate refugia for the species [60].

### 2.5. Conservation Gap Analysis

We conducted a conservation gap analysis to evaluate the effectiveness of existing marine protected areas (MPAs) in safeguarding the distribution ranges of the focal fish species. For this analysis, we used the August 2025 release of the World Database of Protected Areas [61]. Terrestrial protected areas were removed, and only marine polygons were retained. The global MPA shapefile was then clipped to the calibration area of each focal species to ensure that only MPAs within the study region were included. The protected areas in China are not well represented in the WDPA database; therefore, we integrated the data with the National Aquatic Genetic Resources Protection Area zones and the nationwide protected areas in China. To assess coverage, the suitable habitat maps for the current period were overlaid with the MPA polygons, obtained after merging all the shapefiles from the three sources. Conservation efficiency was evaluated by calculating the extent of overlap between the MPAs and the predicted highly suitable, moderately suitable, and low-suitability regions for each species. Area calculations were performed using the Tabulate Area tool in ArcGIS 10.8 (ESRI, Redlands, CA, USA).

## 3. Results

### 3.1. Model Performance

Taking into account the 17 possible combinations of feature classes (FCs) and 31 values of the regularization multiplier (RM), a total of 527 candidate models were generated for each focal fish species. For *C. lucidus*, 525 models were statistically significant, 117 met the omission rate criteria, and only one satisfied the ΔAIC criterion. Consequently, only one model fulfilled all three evaluation requirements. This model, labeled as M_1_F_lqp_Set1, with RM = 1 and lqp feature classes, had optimal parameters and was selected for *C. lucidus* analysis (Table S2). The final ensemble model demonstrated strong reliability, with an AUC of 0.888 and a TSS of 0.725 (Table 1). For *K. punctatus*, all 527 candidate models were statistically significant; six met the omission rate threshold, and two satisfied the ΔAIC criterion. The two models, named according to their feature classes (M_6_F_qh_Set1 and M_8_F_qth_Set1) met all three criteria. Therefore, the final parameterization was set as the average of the two, with RM = 7 using qh feature classes (Table S2). The final model exhibited excellent predictive performance, with a mean AUC of 0.922 and a mean TSS of 0.756 (Table 1). For *C. thrissa*, 341 models were statistically significant, 54 met the omission rate criteria, and six satisfied the ΔAIC criterion. The optimal parameterization selected was RM = 5 with qp feature classes (Table S2). The final model was considered highly reliable, achieving a mean AUC of 0.960 and a mean TSS of 0.920 (Table 1).

### 3.2. The Relative Contribution of Environmental Predictors

The final models for the focal species were constructed using species-specific sets of environmental variables, selected after multicollinearity analysis (Figure 2; Tables S1 and S2). The relative contribution of these variables varied considerably among the three focal species (Figure 2). For *C. lucidus*, dissolved oxygen (O_2_) and salinity (SO) were the most influential predictors. In contrast, chlorophyll-a concentration (Chl) and mixed layer depth (Mlotst) were the strongest predictors for *K. punctatus*, whereas phosphate (PO_4_) and silicate (Si) were the most influential variables in the distribution model of *C. thrissa*.

### 3.3. Potential Distribution Ranges Under the Current Climatic Conditions

The suitable habitats of *C. lucidus* under the present climate scenario were predicted along the coastlines of eastern China and the Korean Peninsula, spanning approximately 22° N to 38° N. Highly suitable regions were concentrated in the coastal waters of the Yellow Sea and East China Sea (Figure 3a), covering an estimated 92,393 km^2^, while moderately suitable regions extended across a larger area of 311,007 km^2^ (Table 2). These suitable habitats fell primarily within the exclusive economic zones (EEZs) of China and South Korea.

In contrast, *K. punctatus* exhibited a broader distributional range, with suitable habitats spanning the coastlines of Japan, Korea, and eastern China. Notably, these regions encompassed the EEZs of China, South Korea, Japan, and Vietnam. Highly suitable regions were predicted in the Yellow Sea, the East and South China Seas, the Central Kuroshio Current, the Sea of Japan, and the Gulf of Tonkin (Figure 3b). The highly suitable areas covered 243,330 km^2^, while moderately suitable regions extended over 707,654 km^2^ (Table 2).

*Clupanodon thrissa* had the smallest calibration area, with suitable habitats predicted mainly in the East China Sea and South China Sea marine ecoregions, within the EEZs of China, South Korea, Japan, and Vietnam. Only a small portion of the eastern sections of these seas was classified as highly suitable, covering approximately 10,869 km^2^, whereas the majority of its predicted habitat fell within moderately and low-suitability regions (Figure 3c; Table 2).

### 3.4. Predicted Shifts in Potential Distribution Ranges Under Climate Change Scenarios

Under future climate scenarios, *C. lucidus* was projected to experience changes in its distributional range. In the best-case scenario (SSP1-2.6), both mid-century (2050–2060) and late-century (2090–2100) projections indicated the largest areas of highly suitable habitat, with predicted ranges of 120,840 km^2^ and 120,390 km^2^, respectively (Table 2). In contrast, SSP3-7.0 and SSP5-8.5 projected smaller highly suitable areas, with the most severe contraction occurring under SSP5-8.5 by late century (2090–2100), when suitable habitat was reduced to 78,823 km^2^. Across all scenarios and projection periods, moderately suitable areas were projected to decline relative to the present (Table 2). Overall, *C. lucidus* was projected to experience more habitat loss than gain, with some stable regions persisting into the future. By late century, projected losses were expected to reach 16% and 18% under SSP3-7.0 and SSP5-8.5, respectively (Figure 4a; Table 3).

In contrast, *K. punctatus* exhibited an opposite pattern, with projected gains in suitable habitat across all future scenarios (Figure 3c and Figure 4b). Highly suitable areas expanded in all scenarios compared to the present, with the largest increases occurring under SSP5-8.5. Moderately suitable habitats also expanded consistently (Table 3). Importantly, several stable regions persisted across scenarios, representing priority areas for conservation. The most substantial gains occurred under SSP5-8.5, with 49% (2050–2060) and 69% (2090–2100) increases in distributional range relative to current levels.

Likewise, *C. thrissa* was projected to undergo the most pronounced expansion in its distributional range. In the mid-century period (2050–2060), highly suitable habitats extended northward into the East China Sea, while moderately suitable areas expanded eastward, encompassing portions of the South Kuroshio Current (Figure 3). Overall, *C. thrissa* showed more gains than losses across all future scenarios, with stable areas persisting alongside substantial new expansions (Figure 4c). In 2050–2060, gains were predicted to reach 45% under SSP1-2.6, 95% under SSP3-7.0, and as high as 230% under SSP5-8.5 (Table 3).

### 3.5. Conservation Status of the Focal Species Due to Climate Change Impacts

**Figure 5:**
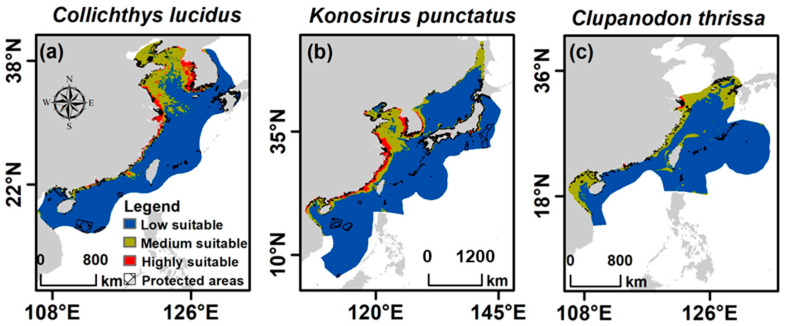
Conservation gaps between current potential distribution ranges and existing marine protected areas (MPAs). Gap analysis results for (**a**) *Collichthys lucidus*, (**b**) *Konosirus punctatus*, and (**c**) *Clupanodon thrissa*.

Most of the current potential distribution ranges of the three focal fish species fell outside existing MPAs (Figure 5; Table S5). Specifically, only 7%, 2%, and 6% of the total distribution ranges of *C. lucidus*, *C. thrissa*, and *K. punctatus*, respectively, were located within MPAs (Table S5). Notably, most observed MPAs were located along the coastlines, particularly in China (Figure 5). However, only 19,576 km^2^ (21%), 4188 km^2^ (39%), and 44,269 km^2^ (18%) of the highly suitable habitats of *C. lucidus*, *C. thrissa*, and *K. punctatus*, respectively, were protected. Notably, only a small portion (0.6%) of the total distribution range for *C. thrissa* was predicted to be highly suitable. Similarly, only a small proportion of the moderately suitable habitats for each species lay within existing MPAs (Table 4).

## 4. Discussion

Our focal species (*C. lucidus*, *C. thrissa*, and *K. punctatus*) are ecologically and economically important fishes in East Asia. Given the increasing threats posed by climate change, detailed knowledge of their distribution patterns and projected shifts is essential for developing effective management strategies. This study provides a comprehensive analysis of the potential suitable habitats of these species under current and future climate scenarios. The findings establish a theoretical basis for designing monitoring frameworks and management measures tailored to the long-term conservation of these species. Furthermore, by identifying priority habitats and regions of stability, our results highlight areas that could serve as potential refugia under climate change. These insights are critical for guiding regional conservation planning and informing policy decisions to safeguard the sustainability of these ecologically important fishes.

Our study revealed that the focal species occupy zones characterized by distinct habitat specificities and are influenced by different climate variables. For *C. lucidus*, dissolved oxygen and salinity were the most influential predictors. Salinity is widely recognized as a primary factor influencing the distribution of species inhabiting estuarine environments, as it determines their physiological tolerance limits [62]. Oxygen availability is also critical, particularly given the documented global decline in dissolved oxygen levels in both open-ocean and coastal waters, which exacerbates habitat loss and physiological stress for marine organisms [63,64]. In contrast, *K. punctatus* and *C. thrissa* were most strongly influenced by chlorophyll and phosphate, respectively, suggesting adaptation to nutrient-enriched waters. Such environments are typically sustained by upwelling systems that transport nutrient-rich waters into the euphotic zone, enhancing phytoplankton biomass and supporting higher trophic levels [65,66]. Importantly, climate-driven intensification and geographic shifts of coastal upwelling, driven by altered atmospheric circulation (the Bakun effect), are expected to expand these nutrient-enriched habitats in the future [67,68].

Projecting species distributions under current and future climate scenarios provides a valuable framework for ensuring long-term species sustainability [69]. Our study revealed clear changes in distribution patterns for the focal fish species across different climate scenarios. For example, *C. lucidus* was projected to undergo range contraction, particularly under the worst-case scenario (SSP5-8.5) by the end of the century, with an estimated 18% loss of suitable habitat. In contrast, the pelagic species *K. punctatus* and *C. thrissa* were projected to expand their distributional ranges in the future. The predicted decline in suitable habitat for *C. lucidus* aligns with broader patterns observed for benthic species, which are often constrained by limited habitat availability and restricted environmental conditions [70,71,72,73]. Conversely, pelagic fishes generally exhibit higher adaptability, and extinction risk is currently considered low for most species [74]. They also display notable changes in seasonal migration patterns [75], which may partly explain the expansion trends projected for *K. punctatus* and *C. thrissa*. Moreover, fish species with faster life histories (e.g., rapid growth and early maturity) tend to respond more rapidly to ocean warming [6]. Such species often shift their distributional ranges quickly [76], influencing ecosystem productivity and trophic dynamics [77]. Pelagic fishes exhibit these traits, making them highly responsive to changes in ocean productivity and thermal niches, thereby facilitating range expansion. The substantial expansion projected for *C. thrissa* suggests that it may emerge as a “climate winner” in the region, benefiting from newly available habitats under future oceanic conditions.

The highly suitable habitats for the three focal fish species were identified along the coastlines of the countries they inhabit. However, these regions fall within national exclusive economic zones (EEZs), leaving the species vulnerable to anthropogenic pressures such as overfishing, pollution, shipping, and coastal development. Our conservation gap analysis further underscores this vulnerability, revealing that only 7%, 2%, and 6% of the total distributional ranges of *C. lucidus*, *C. thrissa*, and *K. punctatus*, respectively, are currently covered by MPAs. This finding aligns with previous studies showing that MPAs often fail to adequately protect marine species and remain insufficient to address the impacts of climate change [78]. Although the global extent of MPAs has increased in recent years [79], significant shortfalls persist in meeting the 2030 targets of the Kunming–Montreal Global Biodiversity Framework. To effectively conserve our focal fish species and other pelagic and benthic taxa in the region, there is a need to expand and reconfigure existing MPAs to better encompass highly suitable habitats, strengthen legal enforcement and market regulation to curb unsustainable exploitation, adopt transboundary conservation strategies that account for species’ distributions across the EEZs of China, Korea, Japan, and Vietnam, and raise public awareness of the ecological and economic importance of conserving these species to sustain biodiversity.

Our study identified several climatically stable regions through range-change analysis, defined as areas that remain suitable across present and future climate scenarios [60]. These regions represent potential climate refugia and should be prioritized for conservation [80]. For the benthic species *C. lucidus*, which is projected to experience range contraction in the Yellow Sea and East China Sea, these stable areas are critical and should be targeted for long-term protection. Incorporating such regions into the design or expansion of marine protected areas would enhance the conservation of this economically important fish. In contrast, the pelagic species, which are projected to expand their ranges, exhibit future distribution shifts that may create new fisheries opportunities for some countries and potential collapses for others, as their ranges extend across multiple EEZs. Such changes could heighten tensions between fisheries management and the governance of shared resources [81]. Developing long-term cooperative strategies to manage these shifting distributions will therefore be essential for balancing conservation and fisheries objectives.

Although our models exhibited very high AUC and TSS values, indicating strong reliability and performance, we acknowledge several limitations that should guide future research. One key limitation is the relatively small number of occurrence records, which can significantly affect model accuracy [82]. For instance, for *C. thrissa*, only 19 occurrence records were available, which may increase uncertainty in predicting its potential distribution. To minimize this limitation, we carefully cleaned the data, removed spatial biases through thinning, and used ensemble modeling to improve predictive robustness despite the limited sample size. Nevertheless, we recommend conducting more extensive field surveys across the full range of these species to enhance data quality, reduce sampling bias, and strengthen the reliability of future models. Another limitation of this study is that future distributions were projected solely using environmental variables, without incorporating anthropogenic pressures such as fishing intensity, pollution, or broader human footprint indices, which are known to influence species distributions [83]. Future research should therefore integrate these socio-ecological variables to provide a more comprehensive understanding of species vulnerabilities. Expanding niche models to include a wider range of ecological and oceanographic drivers, along with human footprint data, would greatly improve their robustness and enhance the environmental realism of future projections [84].

## 5. Conclusions

This study investigated the impacts of climate change on the spatial distributions of three ecologically and economically important fish species in East Asia using an ensemble modeling approach. Our results revealed that different environmental variables drive the distributions of benthic and pelagic species, with *C. lucidus* (benthic) being strongly influenced by dissolved oxygen and salinity. In contrast, *K. punctatus* and *C. thrissa* (pelagic) were primarily shaped by nutrient-related factors. Projections indicated a future contraction of suitable habitats for *C. lucidus*, in contrast to range expansions for the two pelagic species under multiple emission scenarios, highlighting potential shifts in habitat suitability as climate change progresses. The conservation gap analysis further demonstrated that existing MPAs do not adequately encompass the highly suitable habitats of either benthic or pelagic species. This mismatch underscores the urgent need for targeted conservation actions, particularly for *C. lucidus*, which appears most vulnerable.

We recommend strategies such as transboundary conservation planning, the expansion and reconfiguration of MPAs to include priority habitats and strengthened management to mitigate anthropogenic pressures. As climate change continues to reshape marine ecosystems, our study provides critical insights into habitat dynamics, identifies key conservation gaps, and highlights opportunities for adaptive management. These findings contribute not only to the conservation of economically important species in East Asia but also to broader ecosystem-based fisheries management, supporting the sustainable development of regional and transboundary fisheries.

## Figures and Tables

**Table 1:** The distribution records for the focal species (No) and the model performance metrics evaluated using the area under the curve (AUC) and the True Skill Statistic (TSS).

Species	Environment	No	AUC	TSS
*Collichthys lucidus*	Benthic zone	92	0.888	0.725
*Clupanodon thrissa*	Pelagic zone	19	0.960	0.920
*Konosirus punctatus*	Pelagic zone	355	0.922	0.756

Note: The AUC and TSS values shown in the table, represent the mean performance metrics averaged across all models run.

**Table 2:** Predicted suitable habitat projected by the ecological niche models across different time periods in the current and the three future scenarios (2050–2060 and 2090–2100).

Species	Period	Area (km^2^)		
		Highly Suitable	Medium Suitable	Low Suitable
*Collichthys lucidus*	Current	92,393	311,007	1,193,992
	2050–2060			
	SSP126-2050	120,840	283,224	1,193,328
	SSP370-2050	87,548	283,803	1,226,126
	SSP585-2050	86,198	283,632	1,227,563
	2090–2100			
	SSP126-2090	120,390	282,903	1,194,100
	SSP370-2090	81,289	256,321	1,259,782
	SSP585-2090	78,823	250,790	1,267,778
*Konosirus punctatus*	Current	243,330	707,654	3,695,314
	2050–2060			
	SSP126-2050	266,332	813,638	3,566,328
	SSP370-2050	245,495	772,350	3,628,453
	SSP585-2050	281,016	830,937	3,534,345
	2090–2100			
	SSP126-2090	283,632	862,857	3,499,810
	SSP370-2090	258,486	850,295	3,537,517
	SSP585-2090	277,565	960,030	3,408,703
*Clupanodon thrissa*	Current	10,869	331,993	1,420,066
	2050–2060			
	SSP126-2050	55,179	556,545	1,151,204
	SSP370-2050	58,373	562,118	1,142,437
	SSP585-2050	93,529	947,382	722,016
	2090–2100			
	SSP126-2090	30,698	376,946	1,355,284
	SSP370-2090	17,385	310,985	1,434,557
	SSP585-2090	17,814	276,150	1,468,964

**Table 3:** Predicted Area Change in the Distribution Ranges of the Three Focal Fish Species Under Different Climate Scenarios (2050–2060 and 2090–2100).

Species	Time Change	Area Change km^2^				
		Range Expansion	Range Contraction	Stable	% Gain	% Loss
*Collichthys lucidus*	2050–2060					
	Current → SSP126	12,069	42,659	270,512	4	14
	Current → SSP370	3623	25,639	287,533	1	8
	Current → SSP585	4180	28,640	284,532	1	9
	2090–2100					
	Current → SSP126	13,334	26,582	286,590	4	8
	Current → SSP370	7353	51,449	261,723	2	16
	Current → SSP585	8939	57,772	255,399	3	18
*Konosirus punctatus*	2050–2060					
	Current → SSP126	197,734	13,998	536,887	36	3
	Current → SSP370	174,496	18,864	532,021	32	3
	Current → SSP585	270,641	12,476	538,409	49	2
	2090–2100					
	Current → SSP126	242,902	7481	543,404	44	1
	Current → SSP370	249,547	19,165	531,721	45	3
	Current → SSP585	377,590	10,697	540,188	69	2
*Clupanodon thrissa*	2050–2060					
	Current → SSP126	131,815	22,466	268,068	45	8
	Current → SSP370	276,279	49,133	241,401	95	17
	Current → SSP585	668,574	36,164	254,370	230	12
	2090–2100					
	Current → SSP126	77,923	38,736	251,798	27	13
	Current → SSP370	106,048	76,272	214,262	37	26
	Current → SSP585	155,889	77,280	213,254	54	27

**Table 4:** Predicted suitable habitats of the three focal fish species and their overlap with existing marine protected areas (MPAs). The table shows the Area (km^2^) of highly, medium-, and low-suitability regions located inside and outside MPAs, along with their corresponding percentages.

Species	Area (km^2^)								
	Highly Suitable	Outside PA	Inside PA	Medium Suitable	Outside PA	Inside PA	Low Suitable	Outside PA	Inside PA
*Collichthys lucidus*	92,393	72,817 (79%)	19,576 (21%)	311,007	283,609 (91%)	27,398 (9%)	1,193,992	1,132,198 (95%)	61,794 (5%)
*Konosirus punctatus*	243,330	199,061 (82%)	44,269 (18%)	707,654	654,273 (92%)	53,381 (8%)	3,695,314	3,526,489 (95%)	168,825 (5%)
*Clupanodon thrissa*	10,869	6681 (61%)	4188 (39%)	331,993	304,494 (92%)	27,499 (8%)	1,420,066	1,408,885 (99%)	11,181 (1%)

## Data Availability

The data that supports the findings of this study are available in the main text and Supplementary Material of this article.

## Supplementary Materials

The following supporting information can be downloaded at: https://www.mdpi.com/article/10.3390/biology14111476/s1, Table S1: The 16 benthic environmental variables used in the analysis of *Collichthys lucidus*; Table S2: The 24 ocean surface environmental variables (conditions at the top layer of the ocean) used in the analysis of *Konosirus punctatus* and *Clupanodon thrissa*; Table S3: Parameters used to produce candidate models; Table S4: Results of selected models by the kuenm R package. The Feature_classes column denotes the selected feature combination, while the remaining performance statistics represent the median values when multiple best models were selected; Table S5: The total distribution ranges of the focal fish species and the corresponding areas (km^2^) located outside and inside marine protected areas (MPAs).
