# Supplementary material for: Predicting Habitat Suitability and Range Dynamics of Three Ecologically Important Fish in East Asian Waters Under Projected Climate Change
Source: Biology (Basel). 2025 Oct 23;14(11):1476. doi: 10.3390/biology14111476 (PMC12650699; doi:10.3390/biology14111476)
Supplement: Supplementary file 1 [file biology-14-01476-s001.zip › biology-3920413-supplementary.pdf]

List of Supplementary Files

Table S1: The 16 benthic environmental variables used in the analysis of *Collichthys lucidus*.

Serial No.	Code	Environmental variable	Unit	Current	Future
1	thetao*	Ocean temperature	ºC	Y	Y
2	so*	Salinity	-	Y	Y
3	sws*	Sea water velocity	m.s-1	Y	Y
4	swd*	Sea water direction	degree	Y	Y
5	no_3	Nitrate	mmol . m-3	Y	Y
6	po_4	Phosphate	mmol . m-3	Y	Y
7	Si	Silicate	mmol . m-3	Y	Y
8	o_2*	Dissolved molecular oxygen	mmol . m-3	Y	Y
9	dfe*	Iron	mmol . m-3	Y	Y
10	Phyc*	Primary productivity	mmol . m-3	Y	Y
11	Ph	pH	-	Y	Y
12	Terrain characteristics*	Bathymetry	m	Y	
13	Terrain characteristics	Topographic slope	-	Y	
14	Terrain characteristics	Topographic aspect	-	Y	
15	Terrain characteristics	Topographic position index	-	Y	
16	Terrain characteristics	Terrain ruggedness index	-	Y	

An asterisk (*) denotes the variables retained after removing highly correlated predictors. The letter "Y" indicates the availability of a variable for a given time period.

Table S2: The 24 ocean surface environmental variables (conditions at the top layer of the ocean) used in the analysis of *Konosirus punctatus* and *Clupanodon thrissa*.

Serial No.	Code	Environmental variable	Unit	Current	Future
1	thetao	Ocean temperature	ºC	Y	Y
2	so	Salinity	-	Y	Y
3	sws*	Sea water velocity	m.s-1	Y	Y
4	swd*	Sea water direction	degree	Y	Y
5	no_3	Nitrate	mmol . m-3	Y	Y
6	po_4*	Phosphate	mmol . m-3	Y	Y
7	si*	Silicate	mmol . m-3	Y	Y
8	o_2	Dissolved molecular oxygen	mmol . m-3	Y	Y
9	dfe	Iron	mmol . m-3	Y	Y
10	phyc	Primary productivity	mmol . m-3	Y	Y
11	ph*	pH	-	Y	Y
12	chl*	Chlorophyll	mg . m-3	Y	Y
13	sithick*	Sea ice thickness	m	Y	Y
14	siconc	Sea ice cover	Fraction	Y	Y
15	clt	Cloud cover	%	Y	Y
16	mlotst*	Mixed layer depth	m	Y	Y
17	tas	Air temperature	ºC	Y	Y
18	par_mean	Photosynt. Avail. Radiation	E.m-2.day-1	Y	
19	kdpar	Diffuse attenuation	m-1	Y	
20	Terrain characteristics	Bathymetry	m	Y	
21	Terrain characteristics	Topographic slope	-	Y	
22	Terrain characteristics	Topographic aspect	-	Y	
23	Terrain characteristics	Topographic position index	-	Y	
24	Terrain characteristics	Terrain ruggedness index	-	Y	

An asterisk (*) denotes the variables retained for either species after removing highly correlated predictors. The letter “Y” indicates the availability of a variable for a given time period.

Table S3: Parameters used to produce candidate models.

**Parameters**	
Regularization multipliers	0.1, 0.2, 0.3, 0.4, 0.5, 0.6, 0.7, 0.8, 0.9, 1, 2, 3, 4, 5, 6, 8, 10
Feature classes	l, q, p, t, h, lq, lp, lt, lh, qp, qt, qh, pt, ph, th, lqp, lqt, lqh, lpt, lph, lth, qpt, qph, qth, pth, lqpt, lqph, lqth, lpth, qpth, lqpth

Table S4: Results of selected models by the kuenm R package. The Feature_classes column denotes the selected feature combination, while the remaining performance statistics represent the median values when multiple best models were selected.

Species	Regularization_multiplier	Feature_classes	Mean_AUC_ratio	Omission_rate_at_5.	AICc	delta_AICc	W_AICc
Collichthys lucidus	1	Lqp	1.6140	0	1329.98	0.000	0.344
Clupanodon thrissa	5	Qp	1.4748	0	114.98	0.713	0.031
Konosirus punctatus	7	Qh	1.7190	0.04	4425.97	0.370	0.704

Table S5: The total distribution ranges of the focal fish species and the corresponding areas (km^2) located outside and inside marine protected areas (MPAs).

Species	Suitable habitat	Total area (km^2)	
		Outside PA	Inside PA
Collichthys lucidus	1597392	1488624 (93%)	108768 (7%)
Konosirus punctatus	4646298	4379823 (94%)	266475 (6%)
Clupanodon thrissa	1762928	1720060 (98%)	42868 (2%)
